# Thromboprofilaxys With Fondaparinux vs. Enoxaparin in Hospitalized COVID-19 Patients: A Multicenter Italian Observational Study

**DOI:** 10.3389/fmed.2020.569567

**Published:** 2020-11-27

**Authors:** Vincenzo Russo, Giuseppe Cardillo, Giuseppe Vito Viggiano, Sara Mangiacapra, Antonella Cavalli, Andrea Fontanella, Federica Agrusta, Annamaria Bellizzi, Maria Amitrano, Mariateresa Iannuzzo, Clara Sacco, Corrado Lodigiani, Giampiero Castaldo, Pierpaolo Di Micco

**Affiliations:** ^1^Department of Translational Medical Sciences, Monaldi Hospital, University of Campania “Luigi Vanvitelli”, Naples, Italy; ^2^Medylab, Advanced Biochemistry Unit, Naples, Italy; ^3^Emergency Medicine Unit, Marazzini Hospital, Modena, Italy; ^4^Internal Medicine Unit, Moscati Hospital, Avellino, Italy; ^5^Internal Medicine Unit, Frangipane Hospital, Ariano Irpino, Italy; ^6^Internal Medicine Unit, Fatebenefratelli Hospital, Naples, Italy; ^7^Thrombosis and Hemorragic Center, Humanitas Research Hospital and University, Rozzano, Italy

**Keywords:** COVID-19, fondaparinux, enoxaparin, major bleedings, thromboprofylaxis, pulmonary embolism, deep venous thrombosis, acute respiratory distress syndrome

## Abstract

**Importance:** The use of anticoagulant therapy with heparins decreased mortality in hospitalized patients with severe coronavirus disease 2019 (COVID-19). Even if enoxaparin and fondaparinux have the same clinical indication for venous thromboembolism (VTE) prevention; to date, there are no data about the use of fondaparinux in terms of safety, effectiveness, and impact on clinical prognosis among COVID-19 patients.

**Objective:** To evaluate the safety, effectiveness, and clinical impact of VTE prophylaxis with fondaparinux and enoxaparin among COVID-19 patients hospitalized in internal medicine units.

**Design, Setting, and Participants:** This was a retrospective multicenter observation study, including consecutive symptomatic patients with laboratory-proven COVID-19 admitted to internal medicine units of five Italian hospitals from 15th February to 15th March 2020.

**Main Outcomes and Measures:** The primary safety outcome was the composite of major bleeding and clinically relevant non-major bleeding; the primary effectiveness outcome was the composite of all events classified as pulmonary embolism and deep venous thrombosis. The secondary effectiveness outcome included acute respiratory distress syndrome and all-cause death.

**Results:** Among 120 COVID-19 patients enrolled in the study, 74 were taking enoxaparin (4,000 or 6,000 units/day) and 46 fondaparinux (2.5 units/day). No statistically significant difference in demographic and laboratory and clinical characteristics between the two groups has been shown. During a median follow-up of 32 (interquartile range: 14–51) days, the cumulative incidence rates of VTE and bleeding events on pharmacological thromboprophylaxis with heparins were 19% and 8%, respectively. The incidence of both VTE (6.5 vs. 13.5%; *P* = 0.36) and bleeding events (6.5 vs. 4.1%; *P* = 0.68) did not show a significant difference between COVID-19 patients on fondaparinux compared with those on enoxaparin therapy. The regression model for the risk of outcome events according to different VTE prophylaxis drugs did not show significant differences.

**Conclusions and Relevance:** Although these results need confirmation by prospective studies including a larger population, our study provides preliminary evidence of a safe and efficacy use of fondaparinux for VTE prophylaxis in hospitalized COVID-19 patients.

## Introduction

Severe acute respiratory syndrome coronavirus 2 is a highly pathogenic human coronavirus recently recognized as the cause of the coronavirus disease 2019 (COVID-19), which spread rapidly from China to other countries, reaching devastating pandemic proportion (1, 2). The findings of increased D-dimer and fibrinogen levels in COVID-19 patients have prompted questions regarding the coexistence of venous thromboembolism (VTE), exacerbating ventilation–perfusion mismatch; in particular, pulmonary embolism (PE) seems to be prevalent (3). The complex interplay between inflammation and coagulation can significantly affect disease progression, leading to poor outcomes (4). The use of anticoagulant therapy with heparin was shown to decrease mortality in hospitalized patients with severe COVID-19 (5), probably because of its anti-inflammatory and antiviral proprieties (6). Even if enoxaparin and fondaparinux have the same clinical indication for VTE prevention (7), to date, there are no data about the use of fondaparinux in terms of safety, effectiveness, and impact on clinical prognosis among COVID-19 patients. Our study aimed to compare the safety and effectiveness of fondaparinux vs. enoxaparin in VTE prophylaxis among COVID-19 patients hospitalized in internal medicine units; moreover, the clinical impact in terms of acute respiratory distress syndrome (ARDS) development and in-hospital mortality has been evaluated.

## Materials and Methods

### Patient Population

One hundred eighty-six consecutive symptomatic patients with laboratory-proven COVID-19 admitted to internal medicine units of five Italian hospitals from 15th February to 15th March 2020 were retrospectively evaluated for inclusion in the present study. Patients who were taking anticoagulant therapy for any medical reason before COVID-19 diagnosis (*n*: 34) or who experienced recent VTE (*n*: 5), major bleeding (MB), or clinically relevant non-major bleeding (CRNMB) within 30 days of hospital admission (*n*: 6) or with diagnosed VTE at admission (*n*: 21) were excluded from the study. We included hospitalized COVID-19 patients who underwent a VTE prophylaxis regimen according to the current international guidelines (7). During the hospitalization, the clinical status, laboratory examinations, instrumental ultrasound data, therapeutic regimens, the occurrence of PE, deep venous thrombosis (DVT), major (MB) and clinically relevant CRNMBs, acute severe respiratory distress syndrome (ARDS), and all-cause death were assessed.

### Definitions

DVT was defined as a non-compressible venous segment or a substantial increase (4 mm or more) in the diameter of the thrombus during full compression in a previously abnormal segment on ultrasonography. PE was defined as a new intraluminal filling defect on spiral CT or pulmonary angiography or a new perfusion defect of at least 75% of a segment with corresponding normal ventilation (high probability), a new non-high-probability perfusion defect associated with DVT as documented by ultrasonography. MB was defined as fatal bleeding or symptomatic bleeding in a critical area or organ or bleeding causing a fall in hemoglobin level of ≥2 g/dl or more or leading to transfusion of two or more units of whole blood or red cells. CRNMB was defined as overt bleeding, not meeting the criteria for MB but requiring medical intervention (8). ARDS was defined according to the Berlin definition (9).

### Outcomes

The primary safety outcome was the composite of MB and CRNMB; the primary effectiveness outcome was the composite of all events classified as PE and DVT. The secondary effectiveness outcome included ARDS and all-cause death.

### Statistical Analysis

The Anderson–Darling test was used to analyze data normality. Continuous variables were reported using the median and interquartile intervals. Categorical variables were indicated as frequency counts and percentages. Differences in the two groups were evaluated using the two-tailed Fligner–Policello test for continuous data and Fisher's test for categorical variables. The effects of two different pharmacological treatments were tested in univariate analysis for the primary and secondary endpoints by using Cox proportional regressions analysis. Adjusted hazard ratios and 95% confidence intervals were estimated for each endpoint. A two-sided *P* < 0.05 was considered significant for all tests. The net clinical benefit (NCB) was evaluated to obtain an integrated assessment of the anti-thromboembolic and pro-hemorrhagic effects of fondaparinux vs. enoxaparin. NCB was defined as the sum of incidence rates for VTE and bleeding events in the fondaparinux minus the sum of these rates in the enoxaparin group. Analyses were performed using R version 3.5.1 (R Foundation for Statistical Computing, Vienna, Austria).

## Results

We selected 120 hospitalized COVID-19 patients who underwent a VTE prophylaxis regimen according to the current international guidelines. Seventy-four patients were on enoxaparin therapy (4,000 or 6,000 units/day); 46 patients were on fondaparinux (2.5 units/day). The use of fondaparinux and enoxaparin at standard (4,000 units/day) or high dose (6,000 units/day) was based on the patient's VTE risk, estimated by prediction score for risk of VTE (10). All patients showed a Padua Prediction Score for the risk of VTE ≥4. The median follow-up was 32 (interquartile range: 14–51) days. No statistically significant difference in demographic and laboratory and clinical characteristics between the two groups has been shown (Table 1).

**Table 1:** Demographic and laboratory and clinical characteristics of the study population.

**Patients' characteristics**	**Enoxaparin group *N*: 74**	**Fondaparinux group *N*: 46**	***P-*value**
Males, *n* (%)	40 (54%)	24 (52.2%)	0.99
Age (years), median (IQR)	63 (55.3–73.76)	65 (53.6–77.7)	0.78
Hypertension, *n* (%)	36 (48.6%)	22 (47.8%)	0.92
Diabetes Mellitus, *n* (%)	15 (20.3%)	9 (19.6%)	0.88
COPD, *n* (%)	9 (12.2%)	6 (13%)	0.99
CAD, *n* (%)	13 (17.6%)	8 (17.3%)	0.99
CKD, *n* (%)	8 (10.8%)	4 (8.7%)	0.98
DCM, *n* (%)	12 (16.2%)	7 (15.2%)	0.91
PPS, median (IQR)	4 (4–5)	4.5 (4–6)	0.11
Previous stroke/TIA, *n* (%)	6 (8.1%)	3 (6.5%)	0.97
MPAP >40 mmHg, *n* (%)	2 (2.8%)	2 (4.3%)	0.99
D-dimer >500 mcg/dl at admission, *n* (%)	49 (66.2%)	34 (73.9%)	0.49
Fibrinogen >400 mcg/dl at admission, *n* (%)	55 (74.3%)	31 (67.4%)	0.54
Length of hospitalization (days), median (IQR)	31 (14–51)	34 (15–51)	0.90

*CAD, coronary artery disease; CKD, chronic kidney disease; COPD, chronic obstructive pulmonary disease; DCM, dilated cardiomyopathy; PPS, Padua Prediction Score; IQR, interquartile range*.

Thirteen patients experienced incident VTE during the follow-up. The crude incidence rate of VTE was 13.5% (*n*: 10) in enoxaparin vs. 6.5% (*n*: 3) in the fondaparinux group (*P* = 0.36). Six patients experienced bleeding events during the follow-up.

**Figure 1:**
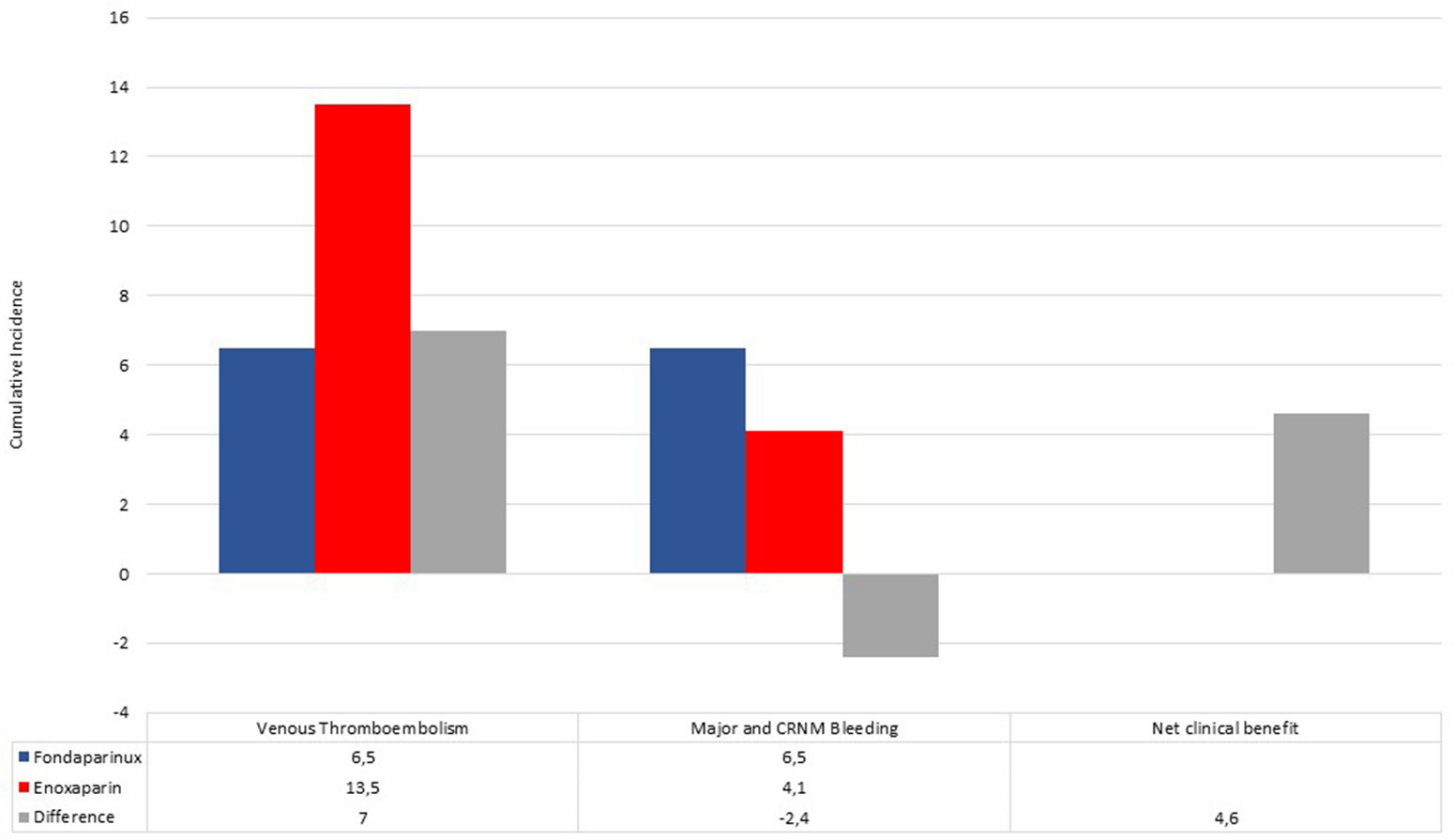
Cumulative incidence of venous thromboembolism events, major and clinically relevant non-major bleedings in fondaparinux (blue bar) and enoxaparin (red bar) recipients. Differences (gray bar) between incidence rates were used to calculate the net clinical benefit (NCB).

The crude incidence rate of bleeding events was 4.1% (*n*: 3) in enoxaparin vs. 6.5% (*n*: 3) in the fondaparinux group (*P* = 0.68). Through these incidence rates, we found a positive NCB of fondaparinux over enoxaparin, equal to +4.6 (Figure 1).

Twenty-one patients developed ARDS during the follow-up. The crude incidence rate of ARDS was 18.9% (*n*: 14) in enoxaparin vs. 15.2% (*n*: 7) in the fondaparinux group (*P* = 0.81). Twelve patients died during the follow-up. The crude incidence rate of all-cause death was 9.5% (*n*: 7) in enoxaparin vs. 10.9% (*n*: 5) in the fondaparinux group (*P* = 0.99). The regression model analysis for pharmacological treatments related to the outcome events is shown in Table 2. The type of VTE prophylaxis drug did not result in a significantly increased risk of VTE, bleeding, ARDS, or in-hospital mortality among COVID-19 patients.

**Table 2:** Incidence and regression model for the risk of outcome events according to different VTE prophylaxis drugs.

**Outcome events**	**Enoxaparin group *N*: 74**	**Fondaparinux group *N*: 46**	**Odds ratio (95% sCI)**	***P***
VTE	10 (13.5%)	3 (6.5%)	2.25 (0.58–8.61)	0.24
DVT	5 (6.8%)	2 (4.3%)	1.59 (0.30–8.58)	0.54
PE	4 (5.4%)	0 (0%)	5.94 (0.31–112.87)	0.24
Bleedings	3 (4.1%)	3 (6.5%)	0.56 (0.11–2.91)	0.50
ARDS	14 (18.9%)	7 (15.2%)	1.30 (0.48–3.51)	0.60
All-cause dead	7 (9.5%)	5 (10.9%)	0.86 (0.25– 2.88)	0.80

*VTE, venous thromboembolism; DVT, deep venous thrombosis; PE, pulmonary embolism; ARDS, acute respiratory distress syndrome*.

## Discussion

The high rate of coagulopathy and VTE among hospitalized patients with COVID-19 has been shown by several studies (11); however, little is still known about the potential association between antithrombotic therapies and COVID-19 clinical presentation or prognosis (12). The World Health Organization recommends the use of pharmacological prophylaxis with heparin for VTE prevention in COVID-19 patients^1^. The once-daily dosing regimen of low-molecular-weight heparins or fondaparinux should be preferred over unfractionated heparin to reduce personal protective equipment use and exposure of healthcare workers (13).

However, despite systematic thrombosis prophylaxis with low-molecular-weight heparins, the incidence of VTE among COVID-19 patients remains remarkably high and well comparable with that in other clinical settings characterized by disseminated intravascular coagulation (14). A recent meta-analysis by Fontana et al. (15) showed that the VTE risk ranges from 4.4 to 8.2% among the overall hospitalized patients with COVID-19; the highest risk, up to 53.8%, has been reported among critically ill patients with COVID-19 pneumonia hospitalized in an intensive care unit. Currently, little is still known about the VTE incidence among COVID-19 patients hospitalized in internal medicine units, and no data on the use of fondaparinux in this clinical setting have been reported yet.

Among our COVID-19 study population, a relatively high cumulative incidence of VTE events (10.8%) during pharmacological thromboprophylaxis with heparins has been showed; however, it was lower than those reported in previous studies from China (25%) and Europe (37%), which included only severe COVID-19 patients admitted to intensive care units (16, 17). The high incidence of VTE events despite the pharmacological thromboprophylaxis with heparins might be explained by the multifactorial genesis of COVID-19-associated coagulopathy. In particular, the excessive release of many inflammatory cytokines and chemokines, such as tumor necrosis factor-α, interleukin (IL)-1, IL-6, and IL-8 (18), and the intense complement activation, with the deposition of the terminal complement complex C5b-9, C4d, and Mannan-binding lectin serine protease 2 in the lungs (19), result in pulmonary microvascular thrombosis, vessel edema and hemorrhagic sequelae. A relatively high cumulative incidence of bleeding events (5%) has been reported, probably due to several prevalent cardiovascular comorbidities, such as diabetes, previous stroke, and hypertension, predisposing *per se* to bleeding events (20). The main finding of our study was that the incidence of both VTE and bleeding events did not show a significant difference between COVID-19 patients on fondaparinux compared with those on enoxaparin thromboprophylaxis; however, fondaparinux showed a higher net clinical benefit compared with enoxaparin. On the other hand, the use of fondaparinux did not show a statistically significant difference in terms of ARDS development and all-cause mortality compared with enoxaparin, with a numerically lower number of both ARDS and death events. These preliminary results support the hypothesis of safe and effective use of fondaparinux, compared with enoxaparin, among COVID-19 patients hospitalized in internal medicine units.

## Study Limitations

The present study has several limitations: the retrospective nature of the analysis; the small number of enrolled patients; the short observational period limited to hospitalization. The study results are preliminary and hypothesis-generating; larger multicenter prospective studies are required to confirm our preliminary findings.

## Conclusion

Our study supports the hypothesis of safe and efficacy use of fondaparinux for VTE prophylaxis in hospitalized COVID-19 patients, justified by a favorable net clinical benefit over enoxaparin.

## Data Availability Statement

The raw data supporting the conclusions of this article will be made available by the authors, without undue reservation.

## Ethics Statement

The ethical committee of University of Campania approved the registry on anticoagulant treatments (751/2019).

## Author Contributions

PD performed study design. GCar, GV, and GCas performed statistical analysis. VR wrote the manuscript. AF, MI, and CL reviewed the draft. AB, MA, SM, AC, CS, FA, and PD selected patients for the study. All authors contributed to the article and approved the submitted version.

## Conflict of Interest

The authors declare that the research was conducted in the absence of any commercial or financial relationships that could be construed as a potential conflict of interest.

## Footnotes

^1^Available online at: https://www.who.int/publications-detail/clinical-management-of-severe-acute-respiratory-infection-when-novel-coronavirus-(ncov)-infection-is-suspected.
